# Lack of Fgf18 causes abnormal clustering of motor nerve terminals at the neuromuscular junction with reduced acetylcholine receptor clusters

**DOI:** 10.1038/s41598-017-18753-5

**Published:** 2018-01-11

**Authors:** Kenyu Ito, Bisei Ohkawara, Hideki Yagi, Hiroaki Nakashima, Mikito Tsushima, Kyotaro Ota, Hiroyuki Konishi, Akio Masuda, Shiro Imagama, Hiroshi Kiyama, Naoki Ishiguro, Kinji Ohno

**Affiliations:** 1 Division of Neurogenetics, Center for Neurological Diseases and Cancer, Nagoya University Graduate School of Medicine, Nagoya, Japan; 2 Departments of Orthopedic Surgery, Nagoya University Graduate School of Medicine, Nagoya, Japan; 3 Departments of Functional Anatomy and Neuroscience, Nagoya University Graduate School of Medicine, Nagoya, Japan

## Abstract

FGF receptor 2 is involved in the formation of the neuromuscular junction (NMJ), but its *in vivo* ligand remains to be determined. Laser capture microdissection of the mouse spinal motor neurons (SMNs) revealed that *Fgf18* mRNA is highly expressed in SMNs in adults. Expression of *Fgf18* mRNA was the highest in the spinal cord at embryonic day (E) 15.5, which gradually decreased to postnatal day 7. FGF18 protein was localized at the NMJs of the tibialis anterior muscle at E18.5 and in adults. *Fgf18*−/− mice at E18.5 showed decreased expressions of the NMJ-specific *Chrne* and *Colq* genes in the diaphragm. In *Fgf18*−/− diaphragms, the synaptophysin-positive areas at the nerve terminals and the acetylcholine receptor (AChR)-positive areas at the motor endplates were both approximately one-third of those in wild-type embryos. *Fgf18*−/− diaphragms ultrastructurally showed abnormal aggregation of multiple nerve terminals making a gigantic presynapse with sparse synaptic vesicles, and simplified motor endplates. In *Fgf18*−/− diaphragms, miniature endplate potentials were low in amplitude with markedly reduced frequency. In C2C12 myotubes, FGF18 enhanced AChR clustering, which was blocked by inhibiting FGFRs or MEK1. We propose that FGF18 plays a pivotal role in AChR clustering and NMJ formation in mouse embryogenesis.

## Introduction

The neuromuscular junction (NMJ) is the synapse that is formed between a spinal motor neuron (SMN) and the skeletal muscle. Induction of the synapse is initiated by binding of neuron-specific agrin to a co-receptor comprised of Lrp4 and MuSK on the muscle endplate. The agrin signal promotes a dynamic network of interacting proteins required for acetylcholine receptor (AChR) clustering^1^. Genetic deletion of the embryonic AChR γ-subunit in myofibers shows decreased intensity of AChR clustering with progressive accumulation of synaptic vesicle proteins^2^. Similar requirement of AChR clustering for appropriate NMJ development has also been reported in knockout mice lacking Lrp4^3^ and agrin^4^. Additionally, growth factors including Wnts, TGF-β, glial cell-derived neurotrophic factor (GDNF), fibroblast growth factors (FGFs), and Rspo2^5^ facilitate AChR clustering and NMJ formation^6^.

FGFs form a family of 22 homologous ligand members in mouse and human. FGFs play roles in signal transductions in different cell types and in different developmental stages through four FGF receptors (FGFR1-4) and mitogen-activated protein (MAP) kinase pathways including mitogen-activated protein kinase kinase 1 (*Map2k1* encoding MEK1 protein)^7–10^. *Fgfr2b* is essential for the formation of presynapse at the NMJ^11^. Some FGF ligands (FGF4, 6, 7, 9, 10, 17, 18, 22, and 23) facilitate aggregation of synaptophysin, neurite elongation, and/or neurite branching in cultured primary spinal motor neurons (SMNs) isolated from chick embryos^12^. However, FGF ligands in the NMJ formation *in vivo* remain to be elucidated. FGF18 is a recently investigated member of FGF family, and is a regulator of osteogenesis^13–15^. In the nervous system, *Fgf18* mRNA is detected in the midbrain and cerebellum by *in situ* hybridization during embryonic development in mice^16,17^. Similarly, FGF18 is detected in the brain by Western blotting up to 8 weeks of age in rats^18,19^. FGF18 has mitogenic activities in cultured astrocytes and microglia^20^, and the neurite branching activity in chick motor neurons^12^. FGF18 additionally has an effect on limb myogenesis by determining the timing of myogenic gene expression in mesenchymal cells^21^. *Fgf18*-deficient (−/−) mice show small lungs, bone abnormalities, and perinatal lethality^14,22^. However, the roles of FGF18 in the formation of embryonic NMJs remain to be dissected.

We found that *Fgf18* mRNA was one of SMN-specific FGFs in adult mice. *Fgf18* mRNA was expressed in the spinal cord, and to a lesser extent in the diaphragm, in mouse embryos. The diaphragm of *Fgf18*−/− embryos showed small synaptophysin- and acetylcholine receptor (AChR)-positive areas at the NMJs. Ultrastructure of the diaphragm NMJs of *Fgf18*−/− embryos revealed gigantic presynapse comprised of abnormally aggregated multiple nerve terminals with decreased densities of synaptic vesicles, and simplified postsynaptic folds. The diaphragm and tibialis anterior muscle of *Fgf18*−/− embryos demonstrated electrophysiologically compromised neuromuscular signal transduction. FGF18 is likely to be one of the essential regulators at the NMJs in mouse embryogenesis.

## Materials and Methods

### Laser capture microdissection and micro array analysis

Laser capture microdissections of SMNs and poster horn cells were previously reported^5^, and the data were deposited in the GEO database with an accession number GSE51122.

### *In situ* hybridization

*In situ* hybridization of the thoracic spinal cord of six-week-old C57BL/6 J mouse was performed as previously described^5^. Briefly, digoxigenin (DIG)-labeled antisense *Fgf18* RNA probe was made using the DIG system (Roche). The sections were incubated for 2 h at room temperature with alkaline phosphatase-coupled anti-DIG antibody diluted to 1:2000 in Buffer 1 [Tris-HCl 100 mM (pH7.5) and NaCl 150 mM] with 0.5% blocking reagent (Roche). The sections were incubated overnight in Buffer 3 [Tris-HCl 100 mM (pH 9.5), NaCl 5 M, and MgCl_2_ 1 M] containing NBT and BCIP (Roche).

### *Fgf18*−/− mice

All mouse studies were approved by the Animal Care and Use Committee of Nagoya University, and were performed in accordance with relevant guidelines. *Fgf18*−/− mice were kindly provided by Dr. Shinji Takada at Okazaki Institute for Integrative Bioscience^14^. Null allele was generated by inserting an *IRES LacZ* sequence at the 16th amino acid downstream of the signal peptide cleavage site in the third exon of *Fgf18*. In all experiments, we used *Fgf18*+/+, +/−, and −/− E18.5 embryos by interbreeding *Fgf18*+/− mice. *Fgf18*−/− progeny resulted in no offspring as previously described^14^. PCR genotyping of embryos were performed with three primers: 5′-CCCAGATGTCATTGGGATAG-3′, 5′-CCCGTGATATTGCTGAAGAG-3′, and 5′-TGAATGGGAGGTCTCTAAGG-3′^14^.

### Quantitative RT-PCR analysis

Total RNA of the spinal cord and the diaphragm of C57BL/6 J and *Fgf18*−/− mice at different embryonic and postnatal days were isolated using Trizol (Thermo Fisher Scientific) or QuickGene RNA cultured cell kit (Kurabo) on QuickGene-800 (FUJIFILM). First strand cDNA was synthesized with ReverTra Ace (Toyobo). cDNA was quantified in triplicates using SYBR Green (Takara) on Light Cycler 480 (Roche). The mRNA levels were normalized for that of *Gapdh*. Primer sequences are shown in Supplementary Table 1.

### Primary SMN culture

We harvested primary motor neurons from the mouse spinal cord as previously described^23^. We took out six embryos at E13.5 from one pregnant *Fgf18*+/− female. Meninges, dorsal root ganglia, and the dorsal half of the spinal cord were removed from the spinal cord of each embryo. We dissociated the ventral half of the spinal cord with 500 μl Sumilon Enzyme Solution. After a centrifugation at 120 × *g* for 4 min, SMNs were suspended in 500 μl Sumilon Dispersion Solution. SMNs were then added with 500 μl Sumilon Isolation Solution, and were again precipitated by centrifugation at 100 × *g* for 5 min. The three Sumilon Solutions were included in the Sumilon Nerve-Cell Dissociation Solutions (Sumitomo Bakelite, 291–78001). SMNs were suspended in 250 μl Sumilon Neuron Culture Medium (Sumitomo Bakelite, 148–09671), and were precipitated by centrifugation at 800 × *g* for 15 min after adding 500 μl OptiPrep (Sigma). SMNs were suspended again in 250 μl Sumilon Neuron Culture Medium, and were precipitated by centrifugation at 70 × *g* for 20 min after adding 4% BSA in PBS. SMNs were suspended in 250 μl Sumilon Neuron Culture Medium with 10 ng/μl of AraC. SMNs were always centrifuged with minimum acceleration/deceleration in a swing rotor. We plated 1 × 10^4^ cells/well in 6–8 wells of a laminin-coated 96-well plate for immunostaining of neurofilament H (a marker for motor neurons) and GFAP (a glial marker), and in 3–5 wells of a laminin-coated 24-well plate for immunostaining of Tau (an axonal marker). The cells were cultured for 2 days. Neurons were fixed with 4.0% formaldehyde in PBS for 15 min at room temperature followed by treatment with 0.1% Triton X-100 for 10 min. After blocking cells with 2% goat serum albumin in PBS, the neurons were incubated overnight at 4 °C with anti-neurofilament H (1:1500, Biolegend, SMI32 801702), anti-GFAP (1:1000, Enzo Life Sciences, EB4), and anti-Tau-1 (1:2000, Millipore, MAB3420) antibodies. The SNMs were washed and incubated with goat anti-mouse Alexa555 secondary antibody (1:250, Abcam, 150114) in 2% goat serum albumin for 2 h. Residual antibodies were removed with repeated washes in PBS. Genotyping of the six embryos revealed that the numbers of *Fgf18*+/+, *Fgf18*+/−, and *Fgf18*−/− embryos were 1, 3, and 2, respectively. We observed that immunostaining for neurofilament H, GFAP, and Tau were similar between the wild-type and *Fgf18*+/− SMNs, but we only compared SMNs derived from one *Fgf18*+/+ and two *Fgf18*−/− embryos. Staining for neurofilament H, GFAP, and Tau was performed in three or more wells for each protein, which required nine or more wells. SMNs from the two *Fgf18*−/− embryos were not mixed throughout the experimental procedures. The number and length of neurites were automatically quantified by the ArrayScan VTI HCS Reader (Thermo Fisher Cellomics).

### Structure and ultrastructure of the NMJ

Diaphragms of *Fgf18*+/+ and *Fgf18*−/− mice at E18.5 were analyzed as previously described^5^. Briefly, the left diaphragm at E18.5 was fixed with 2% paraformaldehyde in PBS at 4 °C, and was rinsed with PBS. The whole-mount left diaphragm was permeabilized with 0.5% Triton X-100 in PBS for 10 min, and then incubated overnight with α-bungarotoxin conjugated with biotin using the Biotin-XX Microscale Protein Labeling Kit (1:800, Invitrogen), anti-peripherin antibody (1:800, Millipore, AB1530), and anti-synaptophysin antibody (1:100, Invitrogen, 180130). After washing, the diaphragm was incubated with Alexa 564-conjugated streptavidin (1:500, Invitrogen) or Alexa 488-conjugated anti-mouse IgG (1:500, Invitrogen). The number and the length of peripherin-positive nerve branches in the diaphragm in five *Fgf18*+/+ and five *Fgf18*−/− mice were quantified by two blinded observers using an FSX100 fluorescence microscope. For quantification of parameters related to clusters of AChR and synaptophysin, confocal laser scanning images were taken with Zeiss LSM710. Two blinded observers used the MetaMorph software (Molecular Devices) to define pixels with AChR-positive signals (red) and synaptophysin-positive signals (green). The area, the signal intensity, the perimeter of the area, and the maximal length of the area were then automatically quantified by MetaMorph^5^.

To analyze the thickness of the diaphragm, sagittal cross sections of frozen diaphragms were fixed with acetone for 10 min at −20 °C, repeatedly washed with PBS, and then covered with PBS containing 2% goat serum for 60 min. The sections were incubated with rabbit polyclonal anti-myosin heavy chain antibody (1:50, Santa Cruz, sc-20641) overnight at 4 °C in a humidified chamber. After the removal of the primary antibody and repeated washes with PBS containing 0.05% Tween-20 (PBS-T), the sections were incubated with the goat anti-rabbit Alexa 488 secondary antibody (1:100, Molecular Probes, A21206) for 1 h.

To analyze the localizations of FGF18 protein, cross sections of the frozen spinal cord and the tibialis anterior muscle at E18.5 and in adults were fixed with acetone for 10 min at −20 °C, washed with PBS several times, and then covered with PBS containing 2% of bovine serum albumin and goat serum for 60 min. The sections of adult spinal cord were incubated with rabbit polyclonal anti-FGF18 (1:50, Santa Cruz, sc-16830) and goat polyclonal anti-choline acetyltransferase (ChAT) antibody (1:100, Millipore, AB144P) overnight at 4 °C in a humidified chamber. After removal of the primary antibody and repeated washes with PBS containing 0.05% Tween-20 (PBS-T), the sections were incubated with the biotin-labeled anti-goat antibody (1:100, Vector, BA-9500), which was followed by a goat anti-rabbit Alexa 488 secondary antibody (1:100, Molecular Probes, A21206) and streptavidin conjugated Alexa 546 (1:800, Thermo Fischer Scientific, S11225) for 1 h. The sections of tibialis muscles were incubated with rabbit polyclonal anti-FGF18 (1:50, Santa Cruz, sc-16830) with α-bungarotoxin conjugated with Alexa594 (1:100, Invitrogen) overnight at 4 °C in a humidified chamber. After the removal of the primary antibody and repeated washes with PBS containing 0.05% Tween-20 (PBS-T), the sections were incubated with a goat anti-rabbit Alexa 488 (1:100, Molecular Probes, A21206) for 1 h.

Ultrastructure of the left diaphragm at E18.5 was analyzed as previously described^5^. Briefly, seven to ten continuous blocks were excised at an interval of 0.2 to 0.3 mm from the central portion of the left diaphragm. As the phrenic nerve could not be traced to its nerve terminal even in wild-type embryos, every second block was stained for cholinesterase using the Ellman method to confirm that the excised blocks indeed included the NMJs. We identified the NMJs by inspecting the entire ultrathin sections of unstained blocks using a JEM-1400 transmission electron microscope. Morphometric analysis of the motor endplate was performed according to Engel and Santa^24^; the following parameters were measured: nerve terminal area in μm^2^, synaptic vesicle density in μm^2^ at the nerve terminal area, area of mitochondria/area of nerve terminal (%), the number of active zones, the diameter of synaptic vesicles, and the width of the synaptic cleft. The postsynaptic fold was defined as a fold in postsynaptic membrane, where the fold depth was more than 70 nm and the width of fold aperture was less than a half of the fold depth. Images were quantified using the ImageJ program (http://imagej.nih.gov/ij/).

### Electrophysiological studies

Phrenic nerve-diaphragm preparations were obtained from four *Fgf18*+/+ and four *Fgf18*−/− embryos at E18.5. Miniature endplate potentials (MEPPs) were recorded and analyzed with the AxoGraph X 1.5.0 software (AxoGraph Scientific) as described previously^25^. We stimulated the sciatic nerve at 2 Hz with a stimulation needle electrode (Inter Medical) and recorded the compound muscle action potentials (CMAPs) of the tibialis anterior muscles using needle electrodes (Inter Medical), which were connected to Neuropack S1 of MEB9704 unit (Nihon Kohden). Data were analyzed with the MEB9704 unit.

### AChR cluster assays

C2C12 myoblasts were seeded on a plate coated with 0.05 μg/μl collagen I (BD Biosciences). C2C12 myoblasts were induced to differentiate into myotubes by culturing cells in DMEM and 2% horse serum for five days. After differentiation, C2C12 myotubes were treated for 12 h with purified agrin (20 ng/ml 550-AG, R&D systems) or recombinant human FGF18 (200 ng/ml C60480, PromoKine) to induce AChR clustering in the presence or absence of an inhibitor for FGFRs (10 μM SU5402, Calbiochem) or an inhibitor for MEK1 (50 μM PD98059, Cell Signaling Technology). DMSO was used to dissolve SU5402 and PD98059, and was also added to the control. Thirty min before fixation in 2% paraformaldehyde, cells were incubated with 10 μg/ml Alexa594-conjugated α-bungarotoxin (Invitrogen) for 30 min to label AChR. Fluorescent images were observed under an Olympus XL71 fluorescence microscope and analyzed with MetaMorph software (Molecular Devices).

### Statistical Analysis

We analyzed the data by unpaired Student’s *t*-test, one-way ANOVA with post hoc Fisher’s LSD using SPSS ver. 21 (IBM). *P* values of 0.05 or less were considered as statistically significant.

## Results

### *Fgf18* gene is expressed in the spinal motor neurons (SMNs) and the diaphragm, and FGF18 protein is localized at the NMJs of tibialis anterior muscle in embryogenesis

To screen for FGF ligands that potentially participate in AChR clustering, we analyzed our previously reported microarray data of ~3,000 laser-capture microdissected SMNs isolated from three 6-week-old C57BL6/J mice^5^. Laser-capture microdissected poster horn cells were used as a control^5^. We found that the expression levels of *Fgf1, Fgf7, Fgf11*, and *Fgf18* were more than 4 times higher in SMNs than in the posterior horn cells (Supplementary Fig. S1A and Supplementary Table 2). Wnt signaling plays an essential role in AChR clustering^6^, and Wnt signaling induces expressions of FGF4, 9, 18, and 20 (http://web.stanford.edu/group/nusselab/cgi-bin/wnt/target_genes). As FGF18 is one of SMN-specifically expressed FGFs and is a downstream target for Wnt signaling in cancer cells^26^, we analyzed the roles of FGF18 in AChR clustering.

**Figure 1:**
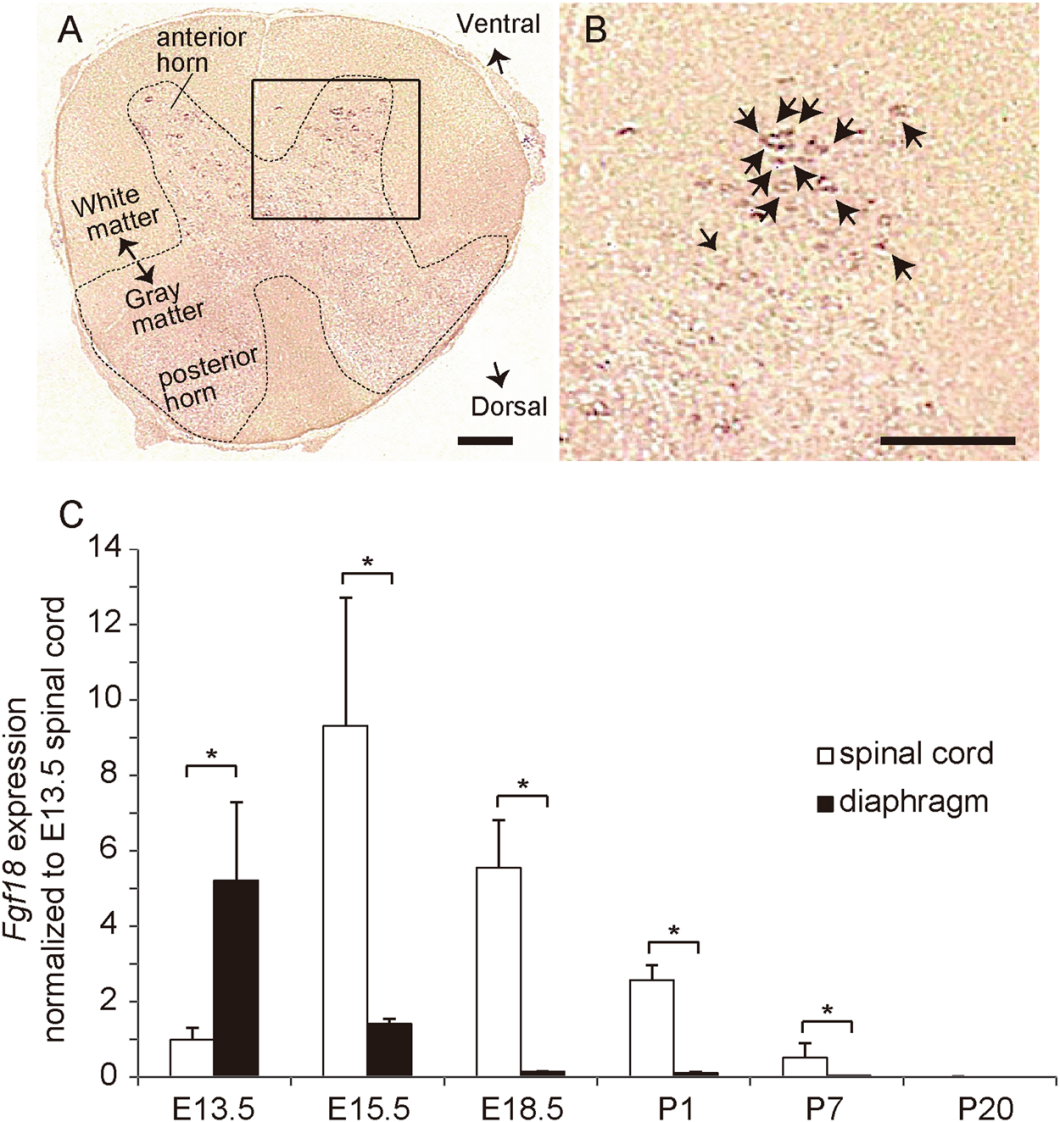
*Fgf18* is highly expressed in spinal motor neurons (SMNs) of the mouse spinal cord. (**A**,**B**) *In situ* hybridization of *Fgf18* of the spinal cord at the middle cervical level of a 6-week-old C57BL6/J mouse. A boxed region is enlarged in (**B**). Arrows point to positive staining for *Fgf18*. Bar = 200 μm. (**C**) Real-time RT-PCR of *Fgf18* in the spinal cord and the diaphragm normalized to that of *Gapdh* and also to the spinal cord at E13.5. Mean and SD (*n* = 3 mice) are indicated. *p* < 0.05 by two-way repeated measures ANOVA. Post-hoc Fisher’s LSD is performed between the spinal cord and the diaphragm, and indicated by *p < 0.05.

We first confirmed specific expression of *Fgf18* in SMNs by *in situ* hybridization (Fig. 1A and B). We traced expression of *Fgf18* in the diaphragm and the spinal cord in embryogenesis (Fig. 1C). Expression of *Fgf18* was detected in the spinal cord from embryonic day 15.5 (E15.5) up to postnatal day 7 (P7), when mature NMJs were formed. Similarly, expression of *Fgf18* was observed to a lesser extent in the diaphragm at E13.5, and almost disappeared at E18.5.

**Figure 2:**
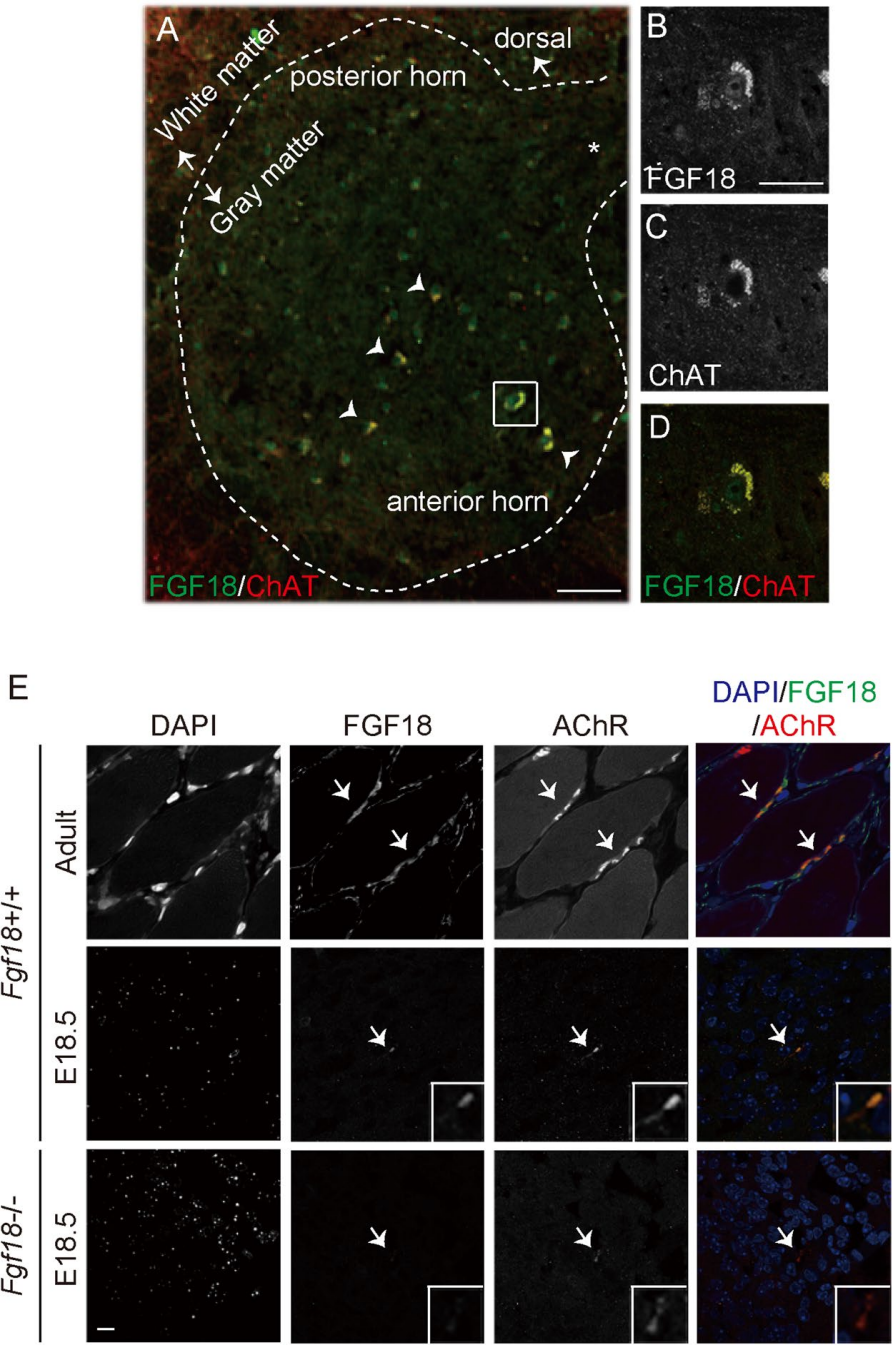
*Fgf18* is expressed in both the spinal cord and the diaphragm. (**A**) Representative immunostaining for FGF18 (green) and choline acetyltransferase (ChAT, red) expressed in SMNs of the adult spinal cord. A region indicated by a white square is enlarged in panels B-D. Note that signals for FGF18 and ChAT are co-localized in SMNs (arrowheads). *Central canal. Bar = 100 μm. (**B**–**D**) High magnification of the squared region in (**A**). Expressions of indicated proteins are shown in (**B**) and (**C**). FGF18 and ChAT are shown in green and red in (**D**). Bar = 10 μm. (**E**) Localizations of FGF18 and AChR in a cross section of the tibialis anterior muscle in adult and of proximal muscles of lower extremities at E18.5. Co-localization of AChR (red) and FGF18 (green) is indicated in the right-most merged images. Arrows point to AChR clusters in each panel. Bar = 20 μm.

We then confirmed colocalization of FGF18 protein and choline acetyltransferase (ChAT), which is a marker for SMNs, in adult SMNs (Fig. 2A–D). In proximal muscles of lower extremities at E18.5 and tibialis anterior muscles in adults, FGF18 was co-localized with AChR clusters (Fig. 2E). FGF18 is thus likely to be generated mostly in SMNs, and is accumulated at the NMJs.

### *Fgf18*-deficient (−/−) mice show reduced expressions of NMJ-specific genes in the diaphragm

**Figure 3:**
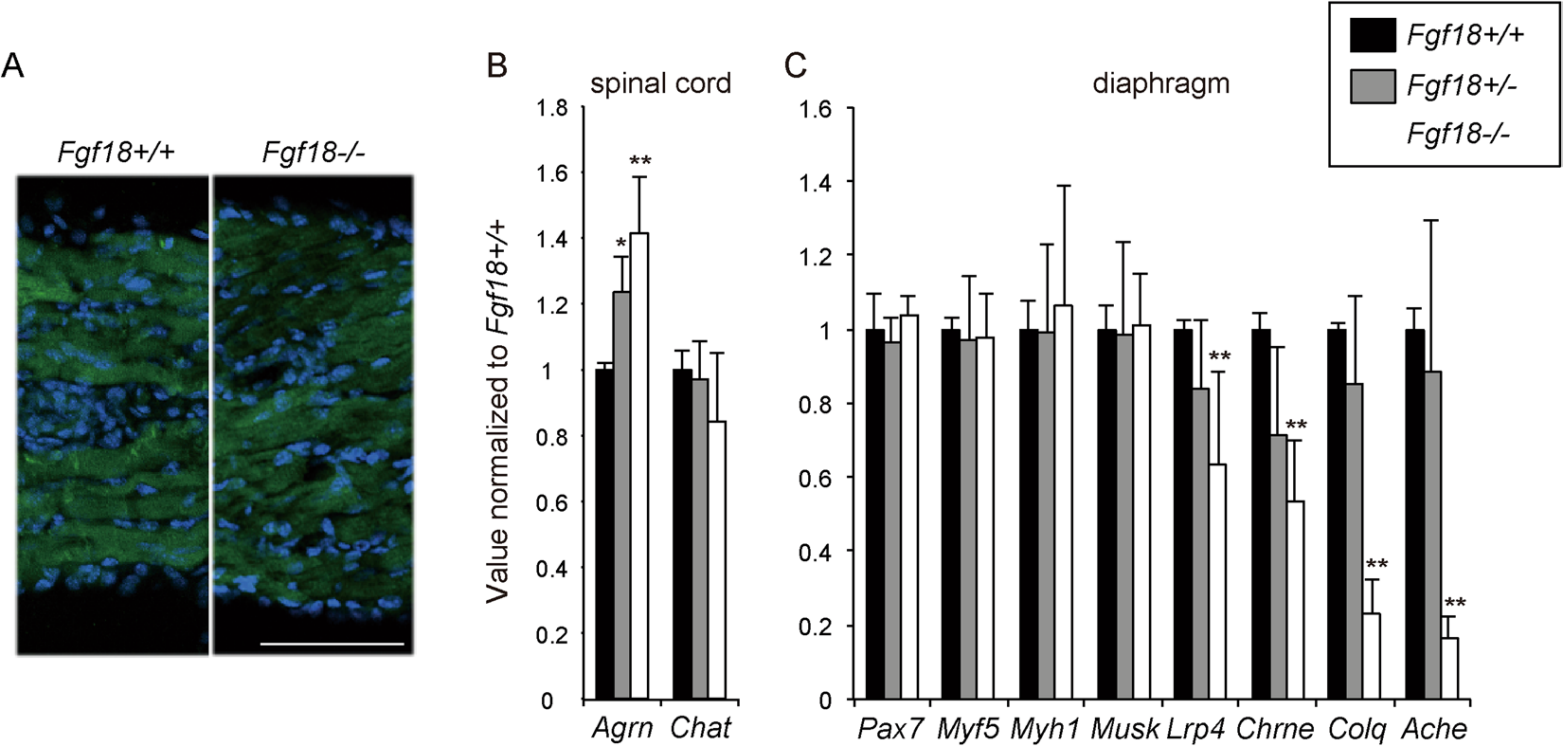
Quantitative real-time RT-PCR of SMN-specific genes in the spinal cord, as well as muscle differentiation marker genes and NMJ-specific genes in the diaphragm, in wild-type *Fgf18*+/+, heterozygous *Fgf18*+/−, and homozygous *Fgf18*−/− mice at E18.5. (**A**) Representative immunostaining with anti-myosin heavy chain antibody (green) for cross sections of the diaphragms of *Fgf18*+/+ and *Fgf18*−/− mice at E18.5. Thickness of the cross sections are blindly measured, and no statistical difference is observed between *Fgf18*+/+ and *Fgf18*−/− mice (Supplementary Table 3). Bar = 500 µm. (**B**,**C**) Gene expressions are normalized to β2-microglobin mRNA and also to *Fgf18*+/+ mice. Bars indicate mean and SD (*n* = 3 mice). *p < 0.05 and **p < 0.01 compared to *Fgf18*+/+ mice by Student’s *t*-test.

Heterozygous *Fgf18*+/− mice were viable, fertile, and morphologically normal. Interbreeding of *Fgf18*+/− mice to generate *Fgf18*−/− progeny resulted in 25% of *Fgf18*−/− embryos. All *Fgf18*−/− mice, however, died perinatally as previously reported^14^. At E18.5, the sizes and thicknesses of the diaphragms (Supplementary Table 3), as well as immunostained signals for myosin heavy chain (Fig. 3A), were similar between *Fgf18*+/+ and *Fgf18*−/− mice. In *Fgf18*+/+, *Fgf18*+/−, and *Fgf18*−/− mice at E18.5, we analyzed expressions of SMN-specific genes (*Agrn* and *Chat*) in the spinal cord (Fig. 3B). We similarly analyzed expressions of NMJ-specific genes (*Musk*, *Lrp4*, *Chrne*, *Colq*, and *Ache*) and muscle differentiation markers (*Pax7*, *Myf5*, and *Myh1*) in the diaphragm (Fig. 3C). In the spinal cord, the expression of *Chat* remained unchanged, whereas the expression of *Agrn* increased with *Fgf18* deficiency. Among the NMJ-specific genes, the expression of *MuSK* remained unchanged, but the expressions of *Lrp4*, *Chrne*, *Colq*, and *Ache* were reduced in homozygous *Fgf18*−/− diaphragms, and to a less extent in heterozygous *Fgf18*+/− diaphragms. In contrast, muscle differentiation markers (*Pax7*, *Myf5*, and *Myh1*) remained unchanged in the *Fgf18*−/− diaphragm.

### Primary SMNs of *Fgf18*−/− mice have short neurites and a reduced number of neurite branches

Being prompted by a previous report that overexpression of FGF18 increases the number of neurite branching in cultured primary chick SMNs^12^, we examined the effect of *Fgf18*-defieincy on neurite elongation and branching in mouse SMNs. We isolated SMNs from *Fgf18*+/+ and *Fgf18*−/− mice at E13.5, when the ventral half of the spinal cord is enriched with SMNs, and primary SMNs can be isolated without being contaminated with other neurons^23^. The isolated SMNs were immunostained for neurofilament H (SMI32) as a marker for motor neurons (Supplementary Fig. S1B). Neurites were immunostained for Tau, and the number and length of neurites were automatically quantified using the ArrayScan VTI HCS Reader. In SMNs of *Fgf18*−/− mice, the neurite lengths were shorter and the number of neurite branch points was less compared to *Fgf18*+/+ mice (Supplementary Fig. S1C–E). Thus, FGF18 has a positive effect on neurite elongation of SMNs, as has been reported in chick embryos^12^.

### *Fgf18*−/− mice show small synaptophysin-positive and AChR-positive areas at the NMJ

**Figure 4:**
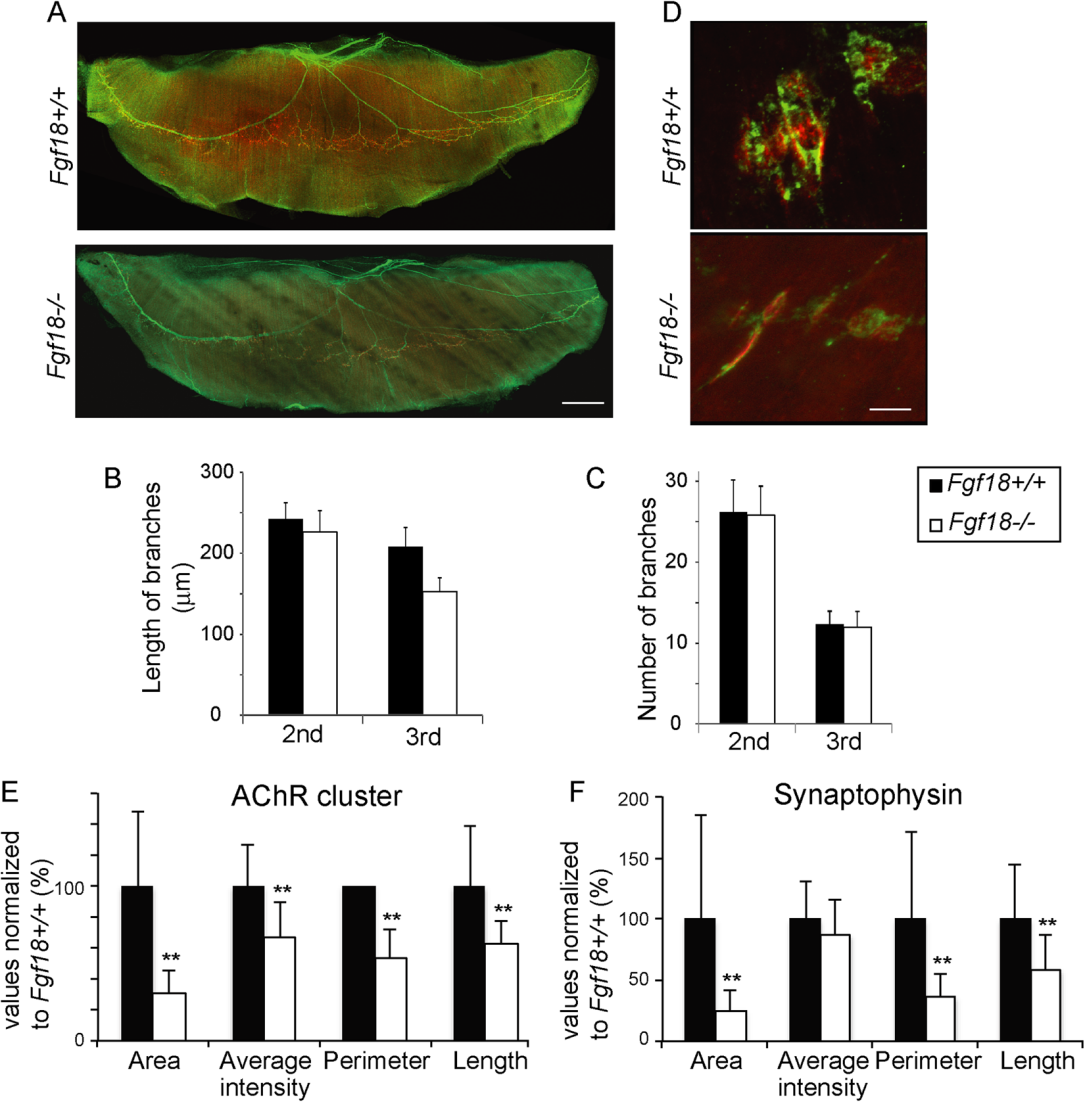
Synaptophysin-positive nerve terminals and AChR-positive motor endplates are small in the E18.5 diaphragm of *Fgf18*−/− mice. (**A**) Representative surface views of the left diaphragms harvested from *Fgf18*+/+ and *Fgf18*−/− mice at E18.5. AChR is stained with Alexa546-conjugated α-bungarotoxin (red) and peripherin/synaptophysin (green) to visual AChR and axons, respectively. Bar = 500 μm. (**B**,**C**) Blinded morphometric analysis of the length (**B**) and the number (**C**) of 2nd and 3rd branches of motor axons. The length and the number of motor axons remain essentially unchanged. Mean and SD (*n* = 15 mice) are indicated. **p < 0.01 by Student’s *t*-test. Bar = 10 μm. (**D**) Representative confocal images of the left diaphragm at E18.5 labeled with an anti-synaptophysin (green) antibody and α-bungarotoxin (red) to visualize the nerve terminals and AChR, respectively. Endplates of the wild-type muscles are mostly ovoid-shaped, whereas the endplates of *Fgf18*−/− muscles are spindle-shaped and weakly stained for AChR. Bar = 10 µm. (**E**,**F**) Blinded morphometric analysis of AChR clusters (**E**) and synaptophysin signals (**F**). The AChR clusters and synaptophysin-positive areas at the NMJ are markedly small at E18.5. Mean and SD (*n* = 15 mice) are indicated. **p < 0.01 by Student’s *t*-test.

In contrast to primary SMNs of *Fgf18*−/− mice, the lengths of the 2nd and 3rd axonal branches, as well as the number of the 2nd and 3rd axonal branches, in the E18.5 diaphragm of *Fgf18*−/− mice were similar to those of *Fgf18*+/+ mice (Fig. 4A–C). All observed AChR clusters were innervated with neuronal axons in *Fgf18*−/− diaphragms (Fig. 4A,D). However, blinded morphometric analysis of the NMJs revealed that the synaptophysin-positive areas at the nerve terminal and the AChR-positive areas at the motor endplates of *Fgf18*−/− diaphragms were both approximately one-third of those of *Fgf18*+/+ diaphragms (Fig. 4E,F).

### The NMJ ultrastructures of *Fgf18*−/− mice show abnormal clustering of nerve terminals with scarce synaptic active zones and simplified endplates

**Figure 5:**
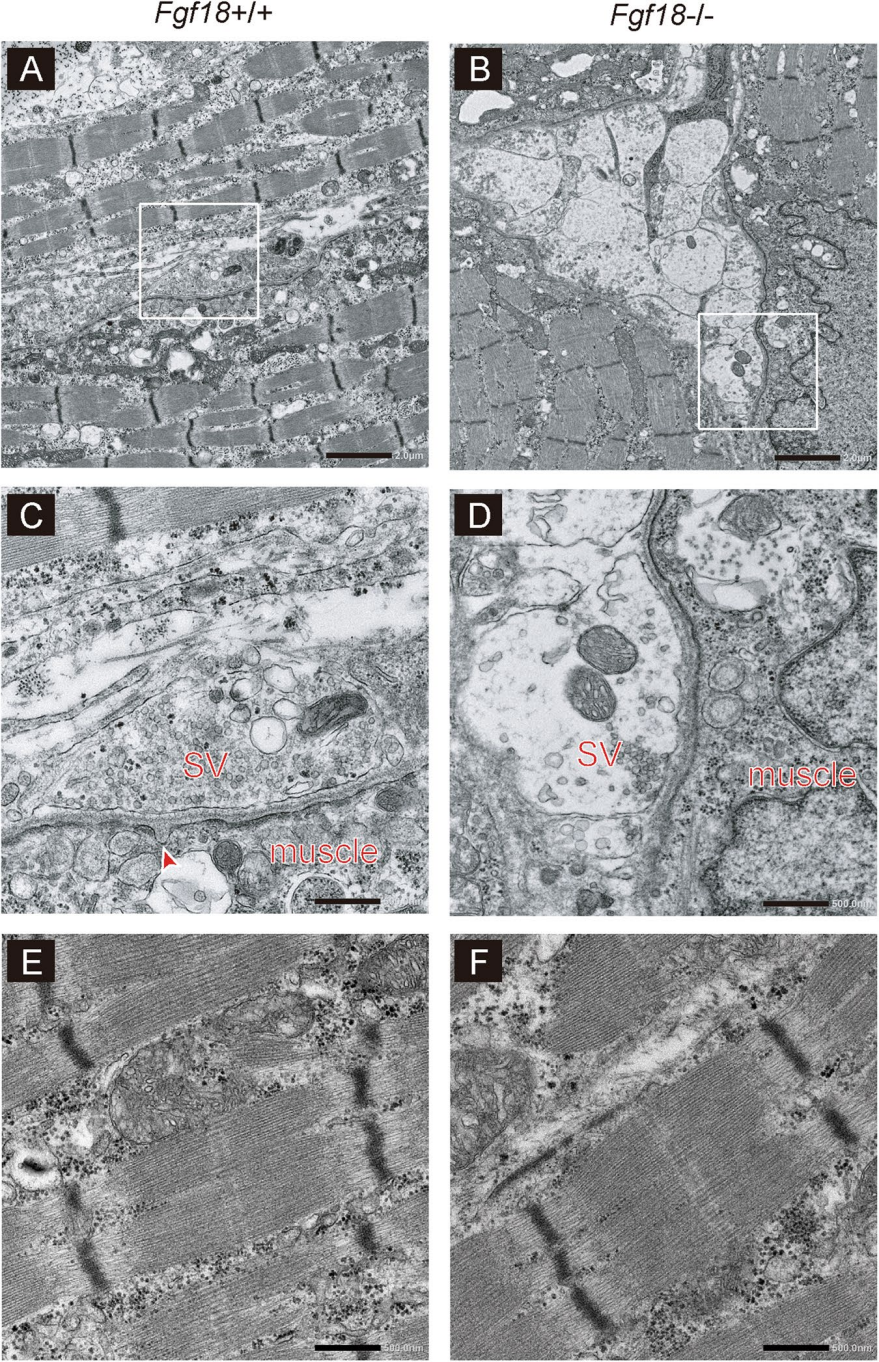
Electron micrographs of the NMJs and muscle fibers of the left diaphragms. (**A**–**D**) Representative electron micrographs of the left diaphragm NMJs of *Fgf18*+/+ and *Fgf18*−/− mice at E18.5. A red arrowhead indicates a postsynaptic fold. SV, synaptic vesicles. Boxed areas in (**A**) and (**B**) are enlarged in (**C**) and (**D**), respectively. Blinded morphometric measurements are shown in Table 1. Bar = 2 μm in (**A**) and (**B**). Bar = 500 nm in (**C**) and (**D**). (**E**,**F**) Representative electron micrographs of the diaphragm muscle fibers of *Fgf18*+/+ and *Fgf18*−/− mice at E18.5. Thickness of muscle fibers at the Z disk in the diaphragms of *Fgf18*+/+ and *Fgf18*−/− mice are 0.82 ± 0.35 and 0.85 ± 0.43 μm (mean and SD, *n* = 5 mice), respectively, with no statistical significance by Student’s *t*-test (not shown). Bar = 500 nm.

We next examined the ultrastructure of the NMJs in the diaphragm of *Fgf18*−/− mice. We found that abnormal aggregation of the nerve terminals made a gigantic presynapse (Fig. 5B). The individual nerve terminals constituting the abnormal aggregates made a synapse with a simplified muscle endplate (Fig. 5B). In addition, synaptic vesicles were sparse, and constituted a synaptic active zone only in a limited area of the nerve terminal (Fig. 5D). All the 17 NMJs observed in four *Fgf18*−/− mice showed similar abnormalities. In contrast, the ultrastructure of muscle fibers in *Fgf18*−/− diaphragm (Fig. 5F) was similar to that of *Fgf18*+/+ diaphragm (Fig. 5E).

Blinded morphometric analysis of the NMJ ultrastructure similarly showed that the presynaptic areas were ~5-fold larger in *Fgf18*−/− mice than *Fgf18*+/+ mice (Table 1). In addition, the densities of synaptic vesicles were ~5-fold lower in *Fgf18*−/− mice. In concordance with the abnormality at the presynaptic regions, the postsynaptic regions of *Fgf18*−/− mice showed significantly fewer junctional folds.

**Table 1:** Parameters of the neuromuscular junction (NMJ) ultrastructure in the diaphragms of *Fgf18*+/+ and *Fgf18*−/− mice.

	*Fgf18*+/+	*Fgf18*−/−	Ratio	*p*
Nerve terminal area (μm^2^)	2.25 ± 0.85	10.34 ± 3.34	4.60	1.86 × 10^−2^
Area of mitochondria/area of nerve terminal (%)	9.88 ± 2.07	5.48 ± 3.33	0.55	5.35 × 10^−1^
Number of mitochondria (/synapse)	1.50 ± 1.3	1.50 ± 1.65	1.00	8.22 × 10^−1^
Diameter of synaptic vesicles (nm)	43.57 ± 7.2 (506)	43.29 ± 4.22 (425)	0.99	9.53 × 10^−1^
Density of synaptic vesicles (/μm^2^)	19.36 ± 3.93	4.2 ± 2.73	0.22	3.43 × 10^−3^
Width of the synaptic cleft (μm)	126.99 ± 17.56 (140)	132.53 ± 19.78 (170)	1.04	1.73 × 10^−1^
Number of active zones (/synapse)	2.71 ± 1.11	1.88 ± 1.04	0.69	8.64 × 10^−3^
Number of postsynaptic folds (/synapse)	1.47 ± 0.54	0.40 ± 0.10	0.27	5.46 × 10^−3^

Blinded morphometric analysis is performed on electron microscopic images of the NMJs in the diaphragms at E18.5. A total of 14 NMJs in 4 *Fgf18*+/+ mice and a total of 17 NMJs in 4 *Fgf18*−/− mice are analyzed. The numbers of analyzed synaptic vesicles and analyzed synaptic clefts are indicated in parentheses, when applicable. Mean and the standard deviation are indicated. Ratio is calculated by dividing the mean value in *Fgf18*−/− mice by that in *Fgf18*+/+ mice. Statistical significance is calculated between *Fgf18*+/+ mice (*n* = 4) and *Fgf18*−/− mice (*n* = 4) with Student’s *t*-test.

### Signal transmission at the NMJ is compromised in *Fgf18*−/− mice

To evaluate the neuromuscular signal transduction in *Fgf18*−/− mice at E18.5, we analyzed the miniature endplate potentials (MEPPs) of the diaphragm on the left side, and the compound muscle action potentials (CMAPs) of the tibialis anterior muscles on both sides. Amplitudes of MEPPs were slightly decreased, and the frequencies of MEPPs were markedly decreased in *Fgf18*−/− mice (Table 2). The markedly decreased MEPP frequency was consistent with the ultrastructurally observed reduced number of active zones (Table 1). Defective neuromuscular signal transmission was also confirmed in abnormally decreased CMAP amplitude measured in the tibialis anterior muscle after repetitive stimulation of the sciatic nerve (Table 2). The abnormal decrement of CMAP in response to repetitive nerve stimulation is a diagnostic hallmark of defective NMJ signal transmission in patients with myasthenia gravis and congenital myasthenic syndromes^27^. These results suggest that FGF18 plays an essential role in signal transmissions at the NMJs *in vivo*.

**Table 2:** Microelectrode measurements and repetitive nerve stimulation in *Fgf18*+/+ and FGF18−/− mice at E18.5.

	Fgf18+/+	Fgf18−/−	p
MEPP amplitude (mV)	3.53 ± 0.33 (n = 16)	2.89 ± 0.70 (n = 18)	0.04
MEPP frequency (sec^−1^)	0.71 ± 1.08 (n = 16)	0.09 ± 0.09 (n = 18)	0.01
Fifth CMAP area (%)	120.5 ± 15.55 (n = 5)	58.4 ± 8.01 (n = 3)	0.0004

Miniature endplate potentials (MEPPs) are recorded from five to six NMJs of the left diaphragm of each of three *Fgf18*+/+ and three *Fgf18*−/− mice at E18.5. Relative areas of the fifth and first compound muscle action potentials (CMAPs) at the 2-Hz stimulation of the sciatic nerve in five *Fgf18*+/+ and three *Fgf18*−/− are indicated. Mean and standard error of mean are indicated. Statistical significance (*p*) is calculated with the Student’s *t*-test.

### Recombinant FGF18 induces AChR clustering in C2C12 myotubes

**Figure 6:**
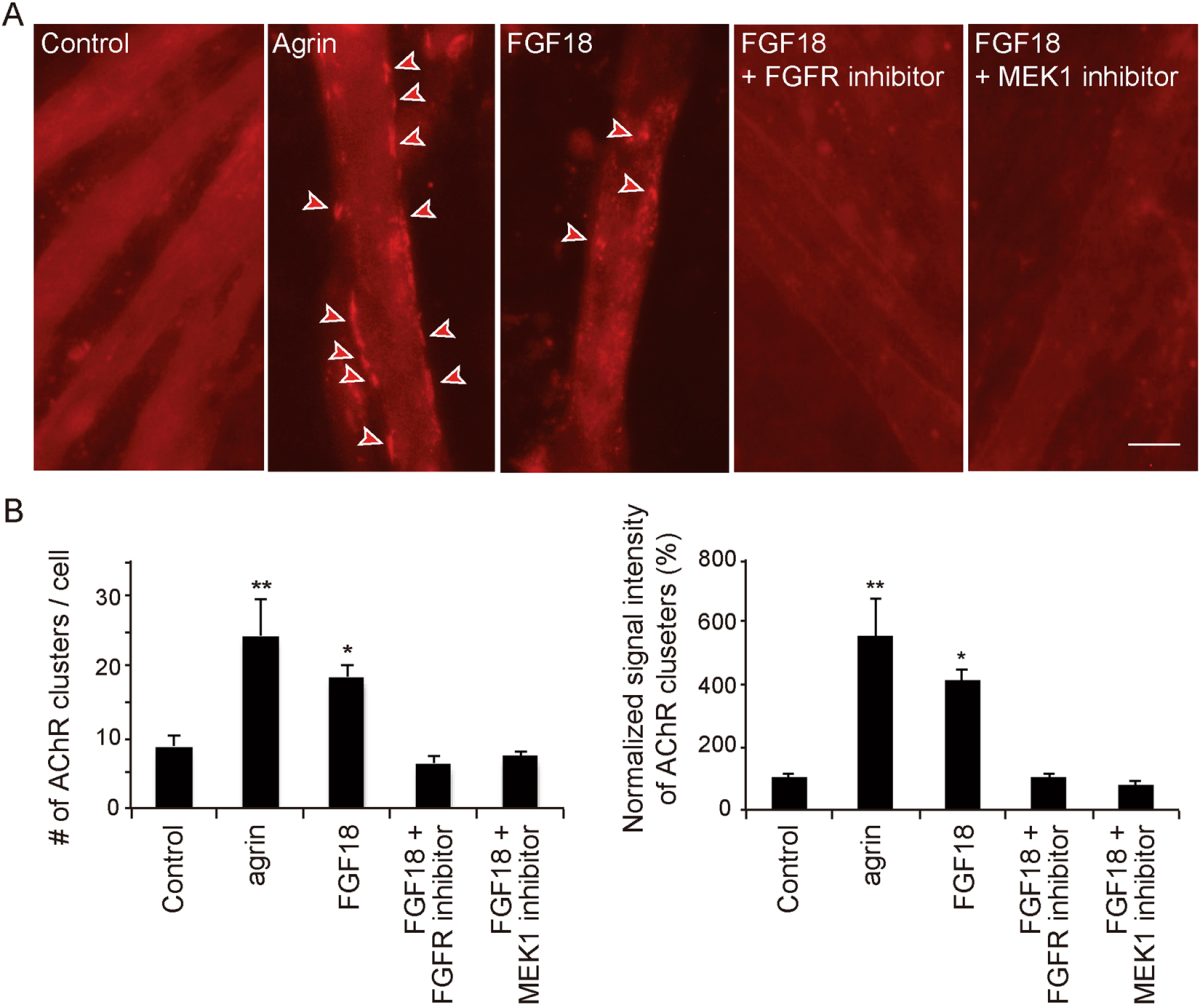
Inhibitors for FGFRs and MEK1 block FGF18-induced AChR clustering. (**A**) BSA (Control), agrin, FGF18, FGF18 with an inhibitor of FGF receptors (FGFRs) (SU5402), and FGF18 with an MEK1 inhibitor (PD98059) are added to C2C12 myotubes. AChR is visualized with Alexa594-conjugated α-bungarotoxin. Arrowheads point to AChR clusters with an axis length of 4 μm or more recognized by the MetaMorph software. Bar = 20 μm. (**B**) Blinded morphometric analysis of AChR clusters. Mean and SE (*n* = 3 independent experiments) are indicated. *p < 0.05 and **p < 0.01 with one-way ANOVA followed by Fisher’s LSD.

Recombinant FGF18 has no significant effects on clustering of synaptic vesicles in primary SMNs derived from chick embryos^12^. However, the effect of recombinant FGF18 on muscle cells has not been studied either in chick or mouse. We thus analyzed the effect of FGF18 on AChR clustering using C2C12 myotubes. Cultured C2C12 mouse myoblasts were differentiated to myotubes by substituting 2% horse serum for 10% FBS for 5 days. Addition of 200 ng/ml of recombinant FGF18 induced AChR clustering in C2C12 myotubes (Fig. 6). Treatment with an inhibitor for FGFRs (SU5402) and an inhibitor for intracellular transducer MEK1 (PD98059) blocked FGF18-induced AChR clustering. These results suggest that exogenous FGF18 has a potential to induce AChR clustering in C2C12 myotubes via FGF signaling.

## Discussion

We analyzed the effects of FGF18 on NMJ formation and AChR clustering. In the nervous system in mice, *Fgf18* is expressed in cerebral cortex at E15.5^28^ and the midbrain-hindbrain boundary region from E8.5 to E10.5^29^. *Fgf18* mRNA is expressed in the nervous system up to P20 in rats^20^. As in the rat brain, expression of *Fgf18* mRNA in the mouse spinal cord peaks at E15.5, and gradually decreases but persists even after birth (Fig. 1C). *Fgf18* mRNA is also expressed in the diaphragm to a lesser extent at E13.5, and gradually disappears at birth (Fig. 1C). As FGF18 is a secreted protein, the origins of FGF18 at the NMJs (Fig. 2E) could not be determined. However, tissue-specific quantification of *Fgf18* mRNA in embryogenesis (Fig. 1C) suggests that FGF18 at the NMJ mostly arises from skeletal muscle at E13.5, which is switched to SMNs at E15.5 and later. In addition to the role of FGF18 at the NMJ presented in this communication, FGF18 regulates the timing of myogenic differentiation of limb mesenchymal cells in chick embryos^21^. Thus, high expression of *Fgf18* at E13.5 in the diaphragm may represent the role of FGF18 in myogenesis, and not in the NMJ formation.

Among the other SMN-specifically expressed FGF ligands (FGF1, FGF7, and FGF11)*, Fgf1* is expressed in various tissues including the nervous system^30^. *Fgf1*−/− mice are indistinguishable from their wild-type littermates at birth, and Nissl-stained sections of *Fgf1*−/− adult neocortex are indistinguishable from those of wild-type mice^31^. *Fgf7* is expressed and functions in morphogenesis including skin^32^, lung^33^, and kidney^34^. *Fgf7* is also expressed in CA3 pyramidal neurons and promotes the organization of inhibitory presynaptic terminals^35^. *Fgf11* is also ubiquitously expressed. *Fgf11* is highly expressed in cancers and enhances cancer growth^36,37^. The role of *Fgf11* in the central nervous system remains to be elucidated. These SMN-specific FGF ligands may or may not have similar functions as observed in FGF18.

A previous study showed that FGF18 protein increased branching points, but had no effect on aggregation of synapsin-positive granules in neurites in cultured SMNs of chick embryos^12^. Primary SMNs derived from *Fgf18*−/− mice at E13.5 demonstrated less neurite elongations and less branch points compared to the *Fgf18*+/+ mice (Supplementary Fig. S1C–E). However, the length and the number of axonal branches in *Fgf18*−/− diaphragm at E18.5 were marginally reduced (Fig. 4A–C). These results suggest that FGF18 has no or minimal effects on neurites of SMNs *in vivo*, or lack of FGF18 in *Fgf18*−/− SMNs may be compensated for by an unidentified molecule including other FGF ligands. Alternatively, FGF18 may exert opposing effects on the neurite branching and elongation at E13.5 and E18.5.

We detected FGF18 protein at the NMJs of adult and embryonic tibialis anterior muscle (Fig. 2E). In *Fgf18*−/− mice at E18.5, gene expressions of muscle differentiation markers (*Pax7*, *Myf5*, and *Myh1*) remained unchanged in the diaphragm (Fig. 3C). In contrast, gene expressions of NMJ-specific molecules (*Chrne* and *Colq*) were decreased even in asymptomatic heterozygous *Fgf18*+/− mice and more in lethal homozygous *Fgf18*−/− mice (Fig. 3B). Reduced expressions of NMJ-specific genes are consistent with markedly reduced synaptophysin-positive nerve terminal areas and AChR-positive areas in *Fgf18*−/− mice (Fig. 4D,E,F). Gigantic presynaptic areas constituted of abnormal aggregation of nerve terminals, restricted synaptic active zones, and simplified endplates with less post-synaptic junctional folds in *Fgf18*−/− NMJs (Fig. 5) are also in accordance with the gene expression profile and the immunohistochemical studies. Markedly decreased MEPP frequency in *Fgf18*−/− diaphragm (Table 2) is consistent with the sparse synaptic vesicles in gigantic presynaptic regions in electron micrographs (Fig. 5 and Table 1). *Fgfr2*−/− mice showed defective NMJ formation characterized by numerous synaptic vesicles extended beyond the synaptic sites^11^. Although the other FGFR genes (*Fgfr1c*, *1b*, *2c*, and *3c*) are also expressed in quadriceps femoris muscle at P5, their functions remain unknown^38^. Among knockout mice of *Fgf* genes, the NMJs were analyzed only for *Fgf5*−/− mice, which, however, showed no NMJ abnormalities^39^. FGFR2 is thus likely to be a receptor for FGF18, but the actual receptor for FGF18 remains to be determined. FGF18 is the first FGF ligand, for which the effects on NMJ formation and AChR clustering have been analyzed.

Among the 22 FGF ligands, FGF8, FGF17, and FGF18 belong to the FGF8 family. These FGFs have high amino acid homologies and share similar biochemical properties^18,40^. Unlike non-secreted FGF family proteins (FGF11, FGF12, FGF13, and FGF14), FGF8 family proteins are secreted and bind to FGFR1-4^41^. FGFRs are engaged in multiple signaling pathways, including extracellular signal-regulated kinases 1 and 2 (Erk1/2), protein kinase Cs (PKCs), phosphoinositide-3 kinase (PI3K), phospholipase Cγ (PLCγ, and signal transduction and activator of transcriptions (STATS). Erk1/2 activation induces expressions of all five AChR subunits in cultured C2C12 myotubes^42^. Muscle-specific double knockout of *Erk1/2* showed fragmented NMJs with faint α-bungarotoxin staining in the sternocleidomastoideus and tibialis anterior muscles in adult mice^43^. Increased PKC activity compromises agrin-induced AChR clustering in cultured C2C12 myotubes^44^. Conversely, lack of PKC θ demonstrates less phosphorylation of AChRδ and ε, polyneuronal innervation, and delayed postsynaptic maturation at the NMJs after P6^45^. PI3K inhibitor, LY294002, blocks recycling of synaptic vesicles at the frog NMJs *in vivo* ^46^. We have shown that inhibitors of FGFRs and MEK1 both block FGF18-induced AChR clustering in C2C12 myotubes (Fig. 6). Similar to C2C12 myotubes, FGF18 enhances osteogenic differentiation through FGFR1 and 2, MEK1, and Erk1/2 in mouse mesenchymal stem cells^47^. AChR clustering induced by FGF18 in C2C12 myotubes is likely to be mediated by activation of FGFRs and MEK1, but other pathways may also be involved in the effect of FGF18 on the NMJ.

## Electronic supplementary material

Supplementary information
