# Supplementary material for: Lack of Fgf18 causes abnormal clustering of motor nerve terminals at the neuromuscular junction with reduced acetylcholine receptor clusters
Source: Sci Rep. 2018 Jan 11;8:434. doi: 10.1038/s41598-017-18753-5 (PMC5765005; doi:10.1038/s41598-017-18753-5)
Supplement: Supplementary file 1 — Supplementary information [file 41598_2017_18753_MOESM1_ESM.pdf]

Lack of Fgf18 causes abnormal clustering of motor nerve terminals at the neuromuscular junction with reduced acetylcholine receptor clusters

Kenyu Ito [1,2,*], Bisei Ohkawara[1,*], Hideki Yagi[1,2], Hiroaki Nakashima[1,2], Mikito Tsushima[1,2], Kyotaro Ota[1,2], Hiroyuki Konishi[3], Akio Masuda[1], Shiro Imagama[2], Hiroshi Kiyama[3], Naoki Ishiguro[2], Kinji Ohno[1]

1) Division of Neurogenetics, Center for Neurological Diseases and Cancer, Nagoya University Graduate School of Medicine, Nagoya, Japan
2) Departments of Orthopedic Surgery, Nagoya University Graduate School of Medicine, Nagoya, Japan
3) Departments of Functional Anatomy and Neuroscience, Nagoya University Graduate School of Medicine, Nagoya, Japan
*These Authors contributed equally to this work.

Corresponding to
Kinji Ohno MD, PhD
Division of Neurogenetics, Center for Neurological Diseases and Cancer, Nagoya University Graduate School of Medicine
65 Tsurumai, Showa-ku, Nagoya 466-8560, Japan
Phone: +81-52-744-2446
fax: +81-52-744-2449
E-mail: ohnok@med.nagoya-u.ac.jp

Key words
Neuromuscular junction; Acetylcholine receptor; Fibroblast growth factor 18 (FGF18)

Supplementary Figure S1. Expressions of FGF ligands in laser-capture microdissected spinal motor neurons (SMNs) in thee 6-week-old mice, and characterization of primary SMNs isolated from *Fgf18*-/- mice at E13.5.

(A) The ratios of mRNA expressions in SMNs and posterior horn cells of FGF ligands (black bars) and FGF receptors (white bars) according to the Affymetrix microarray data[19] are indicated in descending order. Means and SE (n = 3 mice) are indicated. Signal intensity of each gene in each cell type is shown in Supplementary Table 1. *Fgf18* is indicated in bold.

(B) Primary SMNs isolated from *Fgf18+/+* and *Fgf18*-/- embryos at E13.5 are stained with DAPI (blue), SMI32 for neurofilament H (green), and anti-GFAP antibody (green). Representative images are shown. Bar = 50 µm.

(C-E) Quantification of neurites of primary SMNs isolated from one *Fgf18*+/+ and two *Fgf18*-/- embryos at E13.5. Neurite length (C), the number of neurite branches (D), and the number of neurite branches normalized to neurite length (E) are shown by mean and SE (n > 1008 cells). When the numbers of *Fgf18*+/+ and *Fgf18*-/- SMNs are taken into account, **p < 0.01 between *Fgf18*+/+ and *Fgf18*-/- SMNs by Student's *t*-test. One *Fgf18*+/+ and two *Fgf18*-/- embryos hinder us from performing statistical analysis at the embryo level.

Supplementary Table 1. Primer sequences for quantitative RT-PCR

**Target genes**	Forward primers (5’ to 3’)	Reverse primers (5’ to 3’)
mouse *Fgf18*	TATGAACCGAAAAGGCAAGC	AGCCCACATACCAACCAGAG
mouse *Pax7*	AGAGGACGACGAGGAAGGAG	GGGAGGTCGGGTTCTGATT
mouse *Myf5*	TATGAAGGCTCCTGTATCCC	ACGTGCTCCTCATCGTCTG
mouse *Myh1*	CAAGTCATCGGTGTTTGTGG	TGTCGTACTTGGGAGGGTTC
mouse *Musk*	CCTACCCTCAGCCCGAGATTTCTTG	GCCATCATCACTGTCTTCCACGCTC
mouse *Lrp4*	ACCAGGAAATCATTCGCAACAAGC	TGGGGCAGGCACAGGTGTAGTTCTG
mouse *Chrne*	GCCCTGCTTCTCCTGACACTCTTTG	TCGTCCTTGCTGTAGTTGAGCCG
mouse *ColQ*	GTGGTCCTGAATCCAATGAC	GGTTCTTCATGTCTGGGGAG
mouse *Ache*	CTGGGGTGCGGATCGGTGTACCCC	TCACAGGTCTGAGCAGCGTTCCTG
mouse *Gapdh*	ACCCCTTCATTGACCTCAAC	TCCCGTTGATGACAAGCTTC

Supplementary Table 2. Signal intensities (arbitrary unit) of genes encoding FGF ligands and FGF receptors according to microarray analysis of laser-capture microdissected spinal cord samples

Target genes	SMNs		Posterior horn cells		Ratios
	Mean	SD	Mean	SD	
Fgf1	2288	42.14	456	108.90	5.016
Fgf2	90	7.538	171	135.91	0.526
Fgf3	114	14.22	98	23.58	1.164
Fgf4	313	41.77	362	45.99	0.865
Fgf5	451	71.01	263	155.60	1.711
Fgf7	172	205.66	15	6.27	11.875
Fgf8	110	10.41	71	12.92	1.549
Fgf9	2580	187.53	1520	360.60	1.697
Fgf10	330	5.05	542	112.44	0.610
Fgf11	666	31.16	93	8.51	7.125
Fgf12	2748	133.88	1991	171.74	1.380
Fgf13	658	90.00	1890	116.82	0.348
Fgf14	1519	166.51	1796	402.21	0.846
Fgf15	203	29.23	249	21.79	0.818
Fgf16	341	59.31	321	34.46	1.060
Fgf17	139	14.89	115	26.33	1.203
Fgf18	1284	116.30	274	121.21	4.685
Fgf20	58	16.83	47	13.20	1.242
Fgf21	162	2.46	148	36.89	1.091
Fgf22	136	3.76	135	30.31	1.008
Fgf23	236	11.32	254	48.99	0.928
Fgfr1	891	60.15	496	88.14	1.796
Fgfr2	338	44.25	771	289.10	0.438
Fgfr3	335	14.20	806	61.04	0.416
Fgfr4	70	3.72	80	29.34	0.875

Normalized mRNA expression levels of genes for FGF ligands and FGF receptors in SMNs and posterior horn cells of the spinal cords in three 6-week-old C57BL6/J mice according to the transcript-level signal intensities of the Affymetrix microarray data. Ratios of mRNA expressions in SMNs and posterior horn cells are indicated. Ratios are plotted in Fig. 1A in descending order.

Supplementary Table 3. Sizes and thicknesses of diaphragms in *Fgf18*-/- mice

	FGF18 +/+	*FGF18* -/-	*p*
Lateral length (mm)	2.32 ± 0.83 (n = 5)	2.76 ± 0.93 (n = 6)	0.73
Longitudinal length (mm)	5.36 ± 0.74 (n = 5)	5.39 ± 0.36 (n = 6)	0.15
Thickness (μm)	1423.0 ± 94.5 (n = 3)	1465.6 ± 91.6 (n = 3)	0.47

Blinded morphometric analysis is performed on microscopic images of the right diaphragms of *Fgf18*+/+ and *Fgf18*-/- mice at E18.5. The number of quantified diaphragms is shown in parentheses. Mean and the standard deviation are indicated. Statistical significance (*p*) is calculated with the Student's *t*-test.

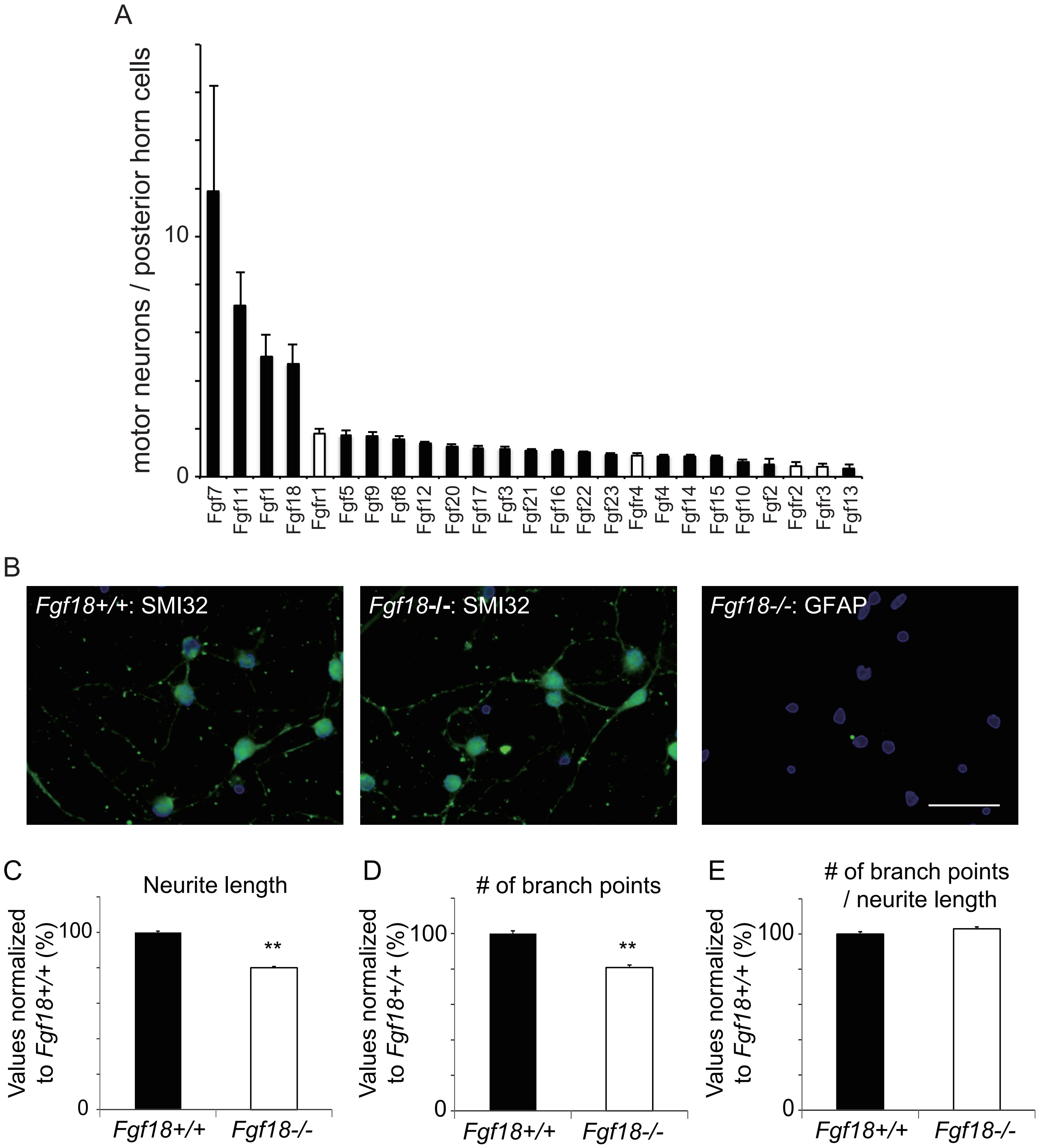
A
motor neurons / posterior horn cells
10
0
Fgf7
Fgf11
Fgf1
Fgf18
Fgfr1
Fgf5
Fgf9
Fgf8
Fgf12
Fgf20
Fgf17
Fgf3
Fgf21
Fgf16
Fgf22
Fgf23
Fgfr4
Fgf4
Fgf14
Fgf15
Fgf10
Fgf2
Fgfr2
Fgfr3
Fgf13
B
Fgf18+/+: SMI32
Fgf18-/-: SMI32
Fgf18-/-: GFAP
C
Neurite length
Values normalized to Fgf18+/+ (%)
100
0
**
Fgf18+/+
Fgf18-/-
D
# of branch points
Values normalized to Fgf18+/+ (%)
100
0
**
Fgf18+/+
Fgf18-/-
E
# of branch points / neurite length
Values normalized to Fgf18+/+ (%)
100
0
Fgf18+/+
Fgf18-/-
